# Isomorphous dinuclear copper(I) complexes bridged by 4-methyl-1*H*-1,2,4-triazole-5-thiol­ate ligands: chloride and bromide analogues

**DOI:** 10.1107/S2056989026007516

**Published:** 2026-07-31

**Authors:** Alfandi Ahmad, Sudthana Saowanit, Nararak Leesakul, Pongsaton Amornpitoksuk, Thitirat Temram, Thitima Rujiralai, Saowanit Saithong

**Affiliations:** a Division of Physical Science and Center of Excellence for Innovation in Chemistry Faculty of Science Prince of Songkla University, Hat-Yai Songkhla 90110 Thailand; b Medical Science Research and Innovation Institute Research and Development Office Prince of Songkla University, Hat-Yai 901120 Thailand; University of Aberdeen, United Kingdom

**Keywords:** dinuclear complex, 4-methyl-1*H*-1,2,4-triazole-5-thione, triphenyl phosphane, isomorphous structure, crystal structure

## Abstract

The title isomorphous copper(I) coordination complexes, bis­(μ-4-methyl-1*H*-1,2,4-triazole-5-thiol­ato)bis­[chlorido­(tri­phenyl­phosphane)copper(II)], [Cu_2_(C_3_H_5_N_3_S)_2_Cl_2_(C_18_H_15_P)_2_], and bis­(μ-4-methyl-1*H*-1,2,4-triazole-5-thiol­ato)bis­[bromido­(tri­phenyl­phosphane)copper(II)], [Cu_2_(C_3_H_5_N_3_S)_2_Br_2_(C_18_H_15_P)_2_], were synthesized from copper(I) halide salts and a mixed-ligand system containing Hmptrz and PPh_3_ in aceto­nitrile (Hmptrz = 4-methyl-1*H*-1,2,4-triazole-5-thione and PPh_3_ = tri­phenyl­phosphine). The asymmetric unit comprises one-half of the dinuclear complex, with each copper(I) center exhibiting a distorted tetra­hedral coordination geometry defined by one halide ion, one phospho­rus atom from PPh_3_, and two μ-S bridging from Hmptrz bridging mol­ecules. The two μ-S bridges [Cu—S—Cu ≃ 70.2°] connect pairs of copper(I) atoms to generate a lozenge-shaped, centrosymmetric Cu_2_S_2_ core. In the extended structure C—H⋯*X* (*X* = Cl or Br) hydrogen bonds link the dimers into a three-dimensional supra­molecular architecture. Hirshfeld surface and two-dimensional fingerprint plot analyses were also carried out to investigate and qu­antify the inter­molecular contacts governing the crystal packing.

## Abstract 2

The title bimetallic complexes are isostructural and feature centrosymmetric rhombic Cu_2_S_2_ cores.

## 1. Chemical context

In recent years, the development of highly efficient luminescent transition-metal complexes has attracted substantial inter­est due to their widespread applications in organic light-emitting diodes (OLEDs), photocatalysis, and chemical sensing. While noble-metal complexes based on Ir^III^ (Kerzig *et al.*, 2019 ▸; Pfund *et al.*, 2020 ▸; Schreier *et al.*, 2022 ▸) and Pt^II^ (Lai & Che, 2004 ▸; Guerchais *et al.*, 2011 ▸; Ma *et al.*, 2013 ▸; Zhong *et al.*, 2013 ▸; Li *et al.*, 2016 ▸) have historically dominated the field, their low crustal abundance and high cost limit their large-scale industrial viability. Therefore, research has shifted significantly toward earth-abundant, cost-effective first-row transition metals. Among these, Cu^I^ complexes have attracted inter­est because their closed-shell 3*d*^10^ electronic configuration suppresses metal-centered non-radiative decay pathways and supports metal–ligand charge-transfer (MLCT) transitions. However, photoexcitation may induce pseudo-Jahn–Teller distortion, resulting in a flattening of the coordination geometry and enhanced non-radiative deactivation in solution. To circumvent these geometric liabilities, research has increasingly focused on dinuclear copper(I) architectures. Utilizing bridging ligands – such as rigid multi-nitro­gen heterocycles or phosphines — secures two copper(I) centers in close spatial proximity. This structural dimerization confers several vital photophysical advantages: (i) enhanced rigidity, which effectively suppresses non-radiative Jahn–Teller distortions (Cao *et al.*, 2026 ▸), (ii) metal–metal inter­actions, driven by the close Cu⋯Cu spatial proximity that modulates the frontier mol­ecular orbitals (Chatterjee *et al.*, 2024 ▸) and (iii) promoted thermally activated delayed fluorescence (TADF) *via* minimized singlet-triplet energy splitting (Li *et al.*, 2020 ▸; Housecroft & Constable, 2022 ▸). Motivated by these advantages, we decided to investigate the synthesis and photophysical properties of a series of copper(I) complexes featuring a mixed-ligand system composed of 4-methyl-1*H*-1,2,4-triazole-5-thione (C_3_H_5_N_3_S; Hmptrz) and tri­phenyl­phosphine (C_18_H_15_P; PPh_3_). In this design, the triazole-thione derivative, Hmptrz, is expected to act as a bridging ligand to link the copper(I) centers into close spatial proximity, while the bulky PPh_3_ co-ligands offer steric inter­ference that stabilizes the coordination sphere. This distinct mixed-ligand environment is hypothesized to enhance structural rigidity, which fundamentally optimizes the luminescent performance for multifaceted technological applications. We now report the syntheses and structures of the title compounds, [Cu_2_(C_3_H_5_N_3_S)_2_(C_18_H_15_P)_2_Cl_2_] (**I**) and [Cu_2_(C_3_H_5_N_3_S)_2_(C_18_H_15_P)_2_Br_2_] (**II**).
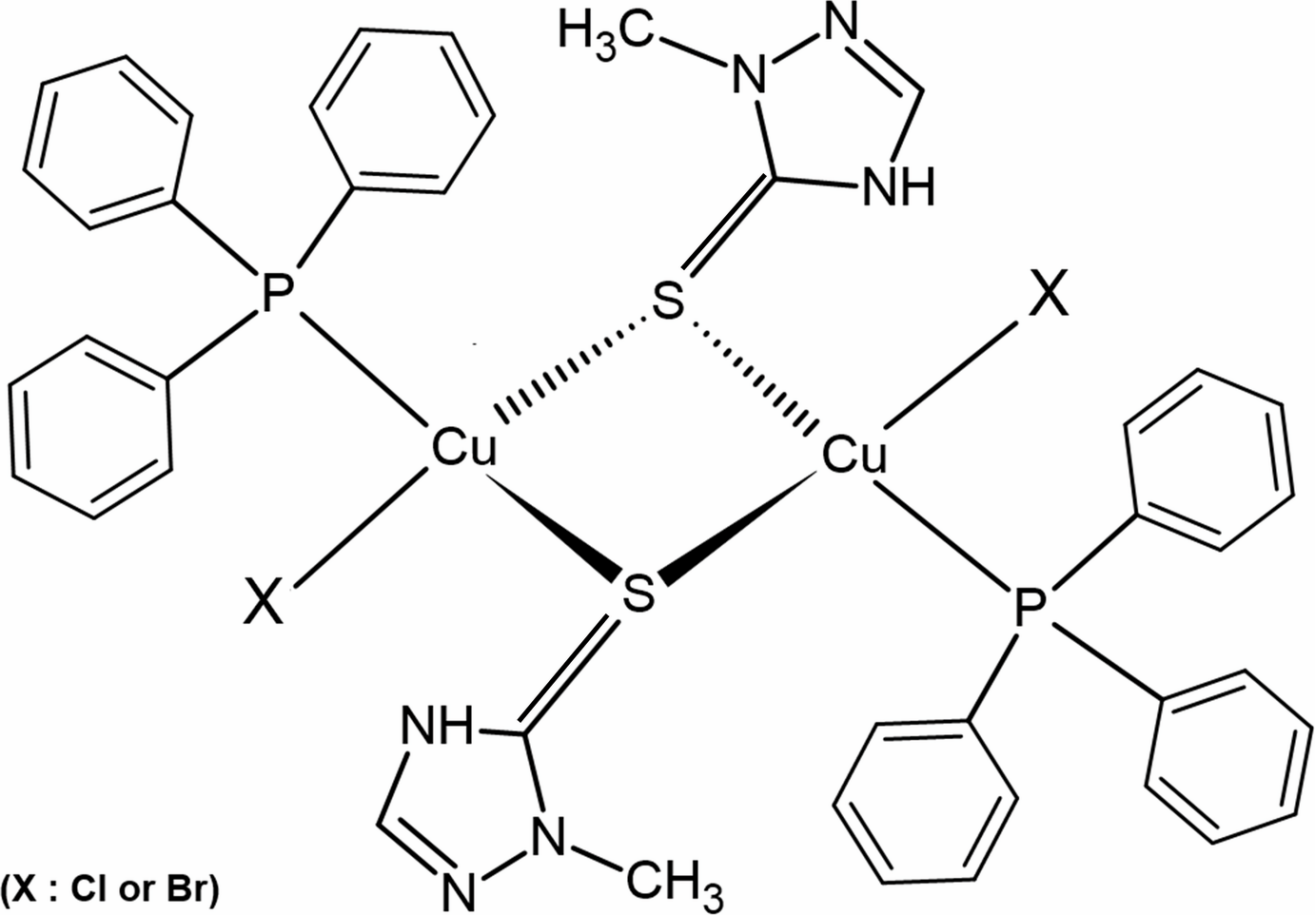


## 2. Structural commentary

**Figure 1:**
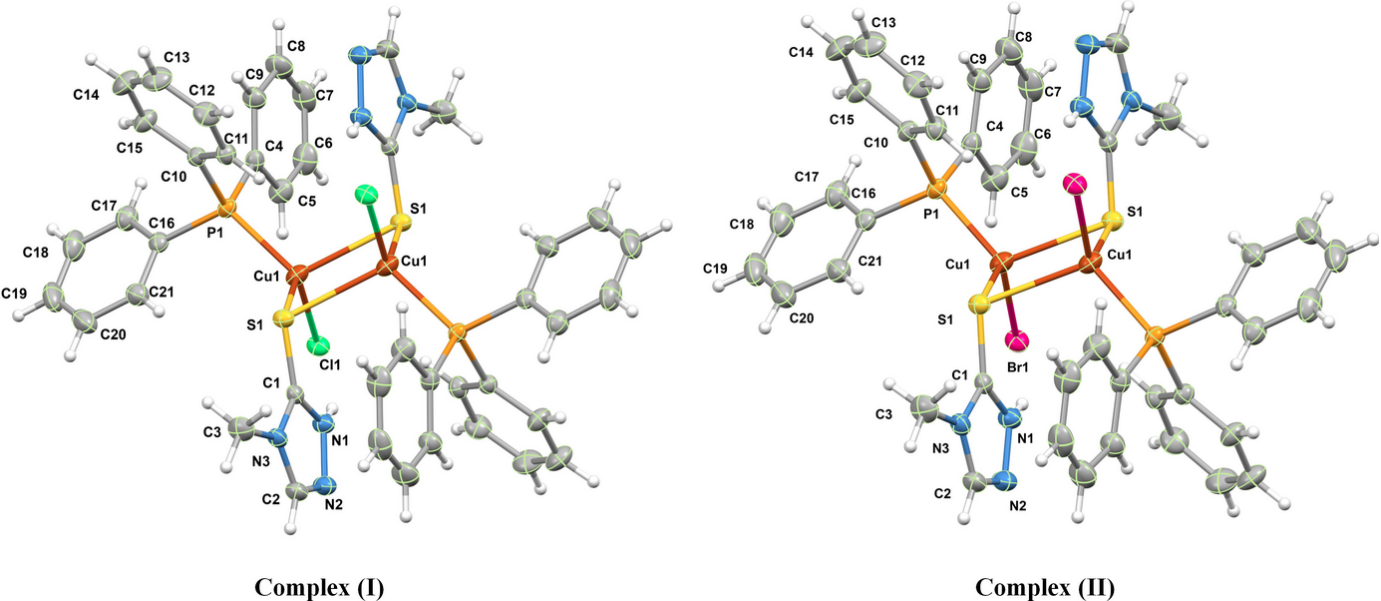
The mol­ecular structures of the title dinuclear complexes (*X*: Cl or Br) with 30% displacement ellipsoids.

**Figure 2:**
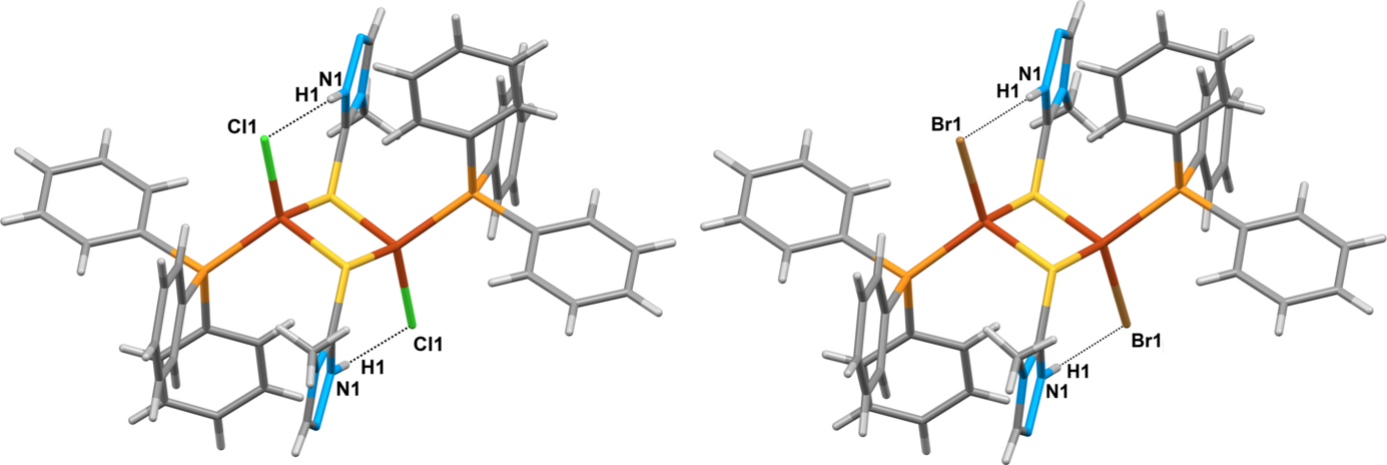
The intra­molecular inter­action, N—H⋯Cl or N—H⋯Br in (**I**) and (**II**), respectively.

Complexes (**I**) and (**II**) are isostructural and crystallize in the triclinic space group *P*ī. The asymmetric unit comprises one Cu^I^ cation, a terminal halide ligand (Cl^−^ or Br^*–*^), one PPh_3_ ligand, and one S-bridging Hmptrz mol­ecule. Crystal symmetry results in the Hmptrz mol­ecules bridging two Cu^I^ cations to form a dinuclear structure. Thus, a centrosymmetric Cu_2_S_2_ core resides about an inversion center, with each Cu^I^ center adopting a pseudo­tetra­hedral geometry. The coordination sphere of the entire mol­ecule contains a central Cu_2_S_2_ core, a pair of terminally bonded halide ions, two S-bridging Hmptrz ligands, and two terminal PPh_3_ ligands. Both complexes exhibit similar coordination geometries around the metal centers, best described as distorted tetra­hedral (Fig. 1 ▸, Tables 1 ▸ and 2 ▸). For example, the bond angles around the copper center range from 95.874 (17)° to 115.482 (18)° for the chloro complex (**I**) and from 96.366 (17)° to 116.06 (2)° for the bromo complex (**II**). The observed Cu–halide distances are 2.3002 (5) Å for Cu1—Cl1 in (**I**) and 2.4284 (4) Å for Cu1—Br1 in (**II**). The Cu1—S1 bond lengths are 2.3245 (5) Å and 2.5781 (5) Å for (**I**) and 2.3268 (6) Å and 2.5501 (7) Å for (**II**). The Hmptrz ligand acts as a bridging motif *via* its S atom, linking two copper centers. The Cu_2_S_2_ core is a common structural feature in this family of complexes (Taylor *et al.*, 1974 ▸) and adopts a distinctive lozenge geometry characterized by alternating short and long Cu⋯S bridging distances. This variation can be attributed to the orbital characteristics and electron-density distribution of the bridging sulfur atom. The S—Cu—S and Cu—S—Cu bond angles are 109.682 (15) and 70.318 (15)° for (**I**), and 109.762 (19) and 70.238 (19)° for (**II**), respectively. These angles are consistent with those commonly observed in related structures (Grifasi *et al.*, 2015 ▸). Given that the sum of the van der Waals radii for two copper atoms is approximately 2.8 Å (Huheey *et al.*, 1993 ▸), the observed Cu1⋯Cu1^i^ [symmetry code: (i) 1 − *x*, 1 − *y*, 1 − *z*) separations of 2.8308 (5) Å for (**I**) and 2.8115 (6) Å for (**II**) indicate a minor Cu⋯Cu inter­action. This is typical for dinuclear Cu^I^ complexes with rhomboidal Cu_2_S_2_ cores, whereas the lozenge geometry generally permits only weak Cu^I^⋯Cu^I^ (cuprophilic) inter­actions (Lobana *et al.*, 2008 ▸, 2009 ▸). Both complexes exhibit a weak intra­molecular N—H⋯*X* (*X* = Cl, Br) hydrogen bond (Fig. 2 ▸, Tables 3 ▸ and 4 ▸).

## 3. Supra­molecular features

**Figure 3:**
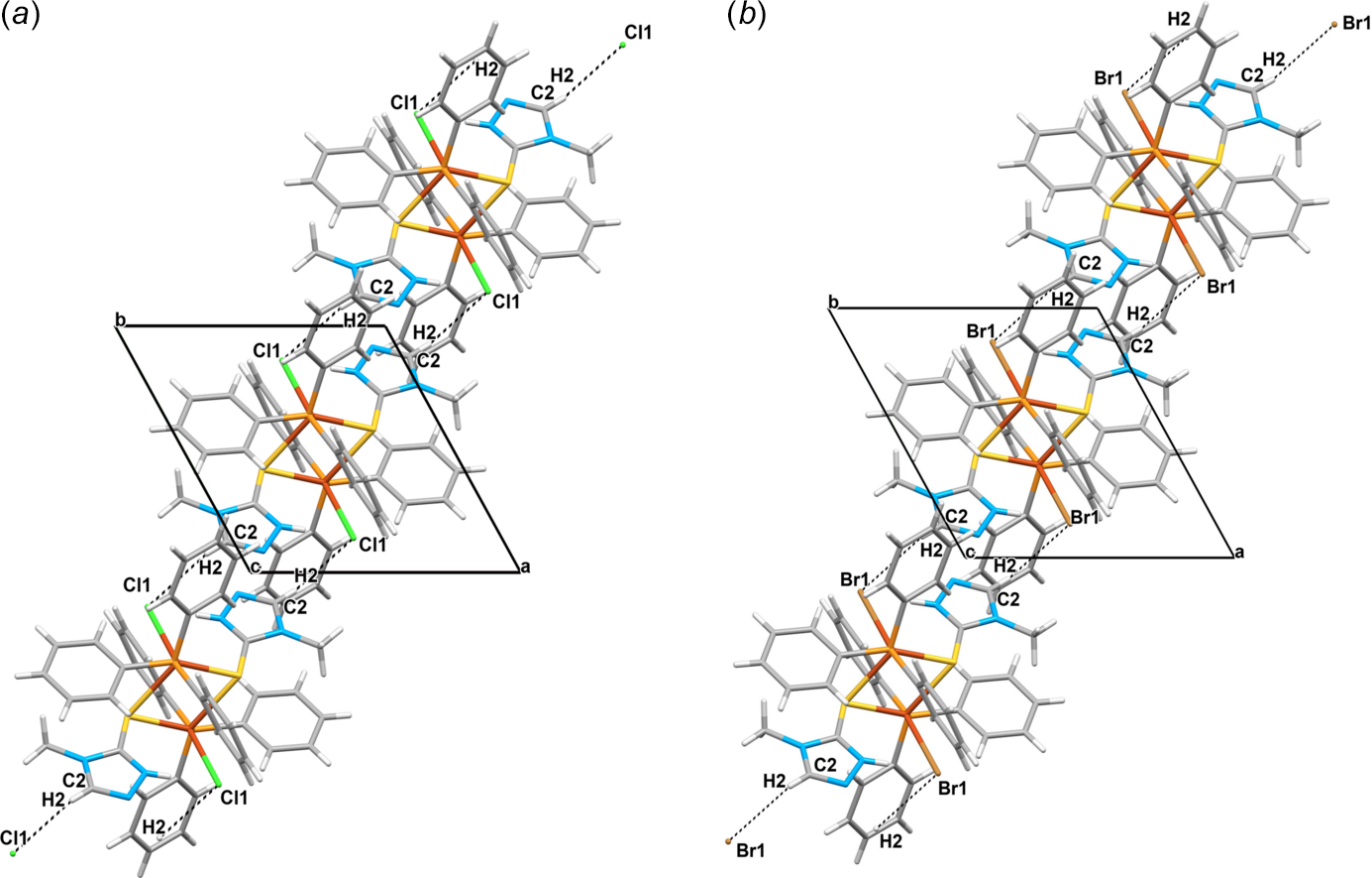
The inter­molecular inter­actions, (*a*) C—H⋯Cl for (**I**) and (*b*) C—H⋯Br for (**II**).

**Figure 4:**
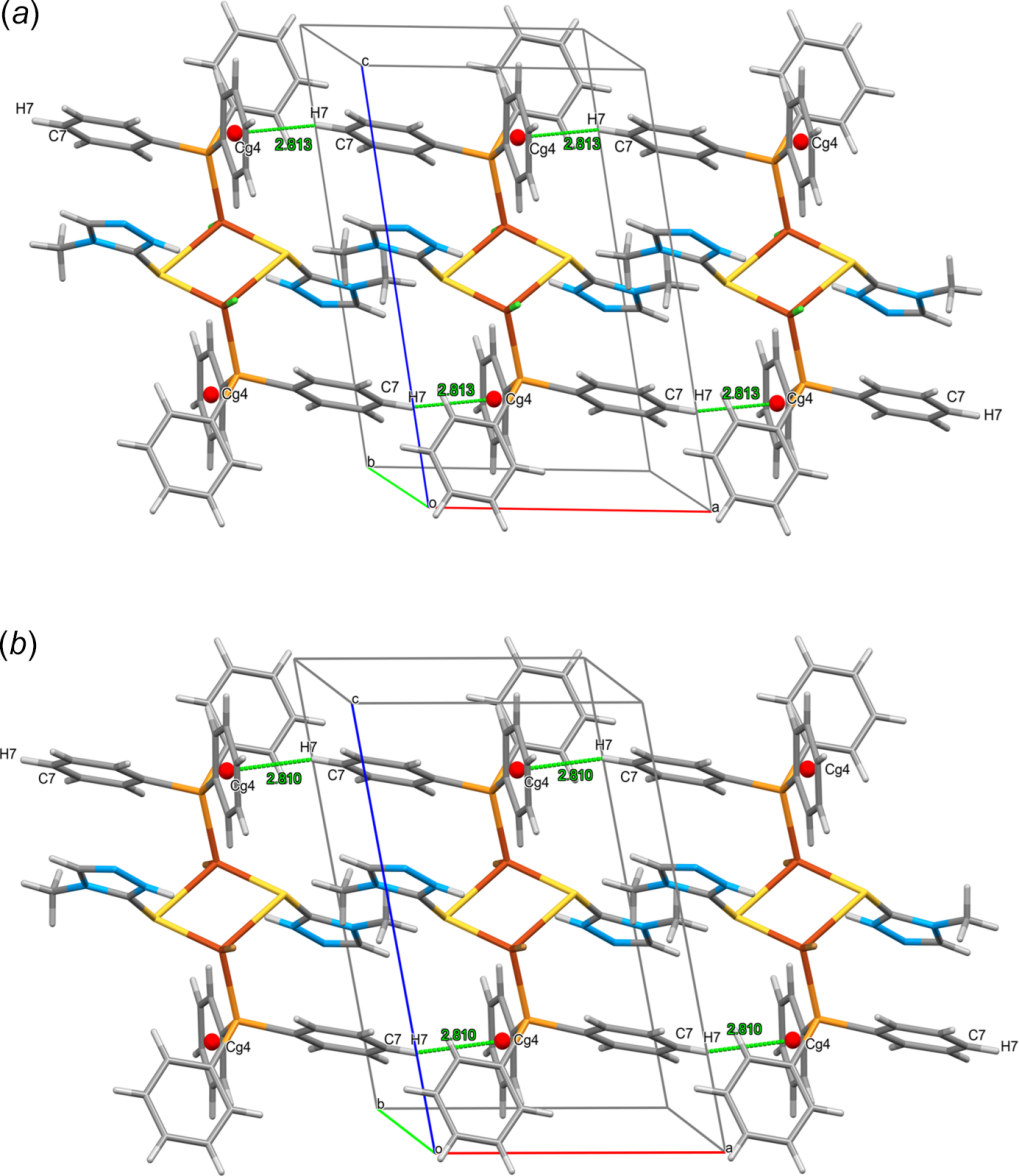
The C—H⋯π inter­molecular inter­actions of (*a*) (**I**) and (*b*) (**II**).

**Figure 5:**
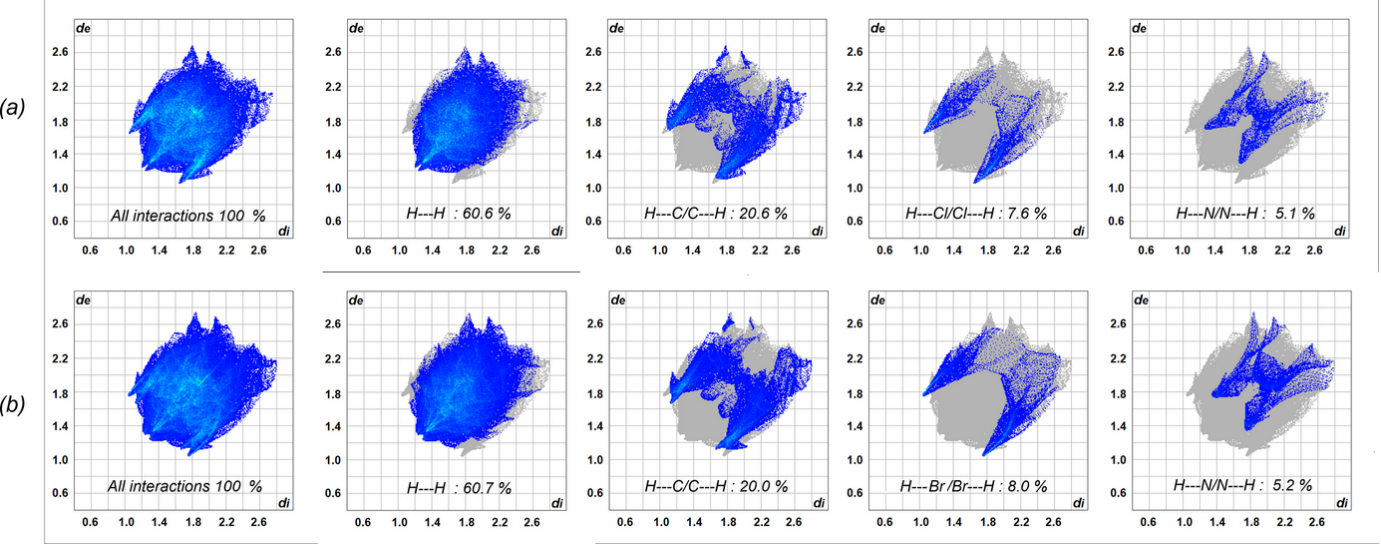
The two-dimensional fingerprint plots for (*a*) (**I**) and (*b*) (**II**) showing all inter­actions and those delineated into H⋯H, H⋯C/C⋯H, H⋯*X*/*X*⋯H and N ⋯H/H⋯N inter­actions (*X*: Cl or Br).

In the extended structures, weak C—H⋯*X* (X = Cl, Br) hydrogen-bonding inter­actions are observed (Tables 3 ▸ and 4 ▸) between adjacent mol­ecules (Figs. 3 ▸*a* and 3*b*). These inter­actions generate supra­molecular chains extending along the diagonal direction of the (110) plane (Fig. 3 ▸). In addition, both complexes exhibit similar C—H⋯π inter­actions. For crystal-packing analysis, a commonly accepted geometrical criterion for a significant C—H⋯π inter­action is an H⋯*Cg* distance shorter than 3.5 Å together with a C—H⋯*Cg* angle greater than 110°. In both structures, the C7—H7 group inter­acts with the centroid of the phenyl ring (*Cg*4 is the centroid of the C10–C15 ring), with H⋯*Cg* distances of 2.81 Å in both (**I**) and (**II**). These inter­actions link neighbouring mol­ecules through their aromatic rings, forming supra­molecular chains propagating parallel to the *a*-axis direction (Fig. 4 ▸). To gain further insight into the crystal packing, a Hirshfeld surface analysis (Spackman & Jayatilaka, 2009 ▸; Turner *et al.*, 2017 ▸) was performed. The corresponding two-dimensional fingerprint plots were used to qu­antify the relative contributions of the various inter­molecular contacts to the crystal packing (Rohl *et al.*, 2008 ▸). The percentage contributions of the individual inter­molecular contacts are presented in Fig. 5 ▸.

## 4. Database survey

A search of the Cambridge Structural Database (CSD, Version 5.47; Groom *et al.*, 2016 ▸) for copper(I) halide complexes containing μ-S-bridging thione ligands and phosphine co-ligands revealed numerous dinuclear Cu^I^ complexes featuring a Cu_2_S_2_ core. Representative examples incorporating a single μ-S-bridging thione ligand include CSD refcodes FOCBII01 and FONBEP (Stergioudis *et al.*, 1987 ▸), JADRUB (Mentzafos *et al.*, 1989 ▸), IVEREG (Aslanidis *et al.*, 2004 ▸), ETUCUN02, HOMYOY, MEPVIN01, HOMZAL, HOMYAK, HOMYEO and HOMYUE (Bowmaker *et al.*, 2009*b* ▸), together with WUDFUX and WUDGEI (Bowmaker *et al.*, 2009*a* ▸).

A number of closely related dinuclear Cu^I^ complexes containing μ-S-bridging thione ligands in combination with phosphine ligands have also been reported. These include JADHAX and VIPBIF (Karagiannidis *et al.*, 1989 ▸, 1990 ▸), JITFOH (Hadjikakou *et al.*, 1991 ▸), YEZDOW (Aslanidis *et al.*, 1994 ▸), GADHID (Aslanidis *et al.*, 2002 ▸), WALROR (Hadjikakou *et al.*, 2005 ▸), CITFAN (Mamais *et al.*, 2008 ▸), TOMFIM (Papazoglou *et al.*, 2014 ▸), ADIGUS (Varna *et al.*, 2016 ▸) and TEQQAK (Karasmani *et al.*, 2018 ▸). Despite differences in the nature of the thione, phosphine and terminal halide ligands, these complexes share a common Cu_2_S_2_ core and a distorted tetra­hedral coordination geometry around the Cu^I^ centres. The title compounds therefore belong to this family of dinuclear Cu^I^ complexes and expand the structural diversity of μ-S-bridged copper(I) phosphine compounds.

## 5. Synthesis and crystallization

Synthesis of (**I**)

4-Methyl-4*H*-1,2,4-triazole-3-thiol (0.175 g, 1.52 mmol) was dissolved in 20 ml of aceto­nitrile and heated to 343 K. Copper(I) chloride (0.150 g, 1.52 mmol) was then added, and the reaction mixture was stirred continuously at 343 K for 3 h. Subsequently, a solution of tri­phenyl­phosphine in aceto­nitrile (10 ml) was added dropwise, and the mixture was stirred for a further 2 h at the same temperature. The reaction mixture was filtered to remove the insoluble solid. The clear filtrate was left to evaporate slowly at room temperature. After several days, colorless crystals of (**I**) suitable for X-ray diffraction were obtained. The crystals were separated and dried. The crystals obtained consisted of a mixture of crystalline products. Therefore, neither the isolated yield nor the elemental analysis could be determined for the target complex. Nevertheless, a suitable single crystal was selected for single-crystal X-ray diffraction analysis.

Synthesis of (**II**)

A mixture of copper(I) bromide (0.150 g, 1.05 mmol) and 4-methyl-4*H*-1,2,4-triazole-3-thiol (0.120 g, 1.04 mmol) was dissolved in 20 ml of aceto­nitrile and heated to 343 K and stirred continuously for 3 h. In a separate vessel, tri­phenyl­phosphine (0.550 g, 2.10 mmol, 2 equiv.) was dissolved in 10 ml of aceto­nitrile and added dropwise to the reaction mixture. The resulting solution was stirred at 343 K for an additional 2 h and subsequently filtered to remove any insoluble material. The clear filtrate was allowed to evaporate slowly at room temperature, affording colorless crystals of (**II**) after several days. The crystals were collected by filtration and dried. Yield: 0.4315 g (79.5%). m.p. 217.7–218.7 °C. Analysis calculated for C_42_H_40_Br_2_Cu_2_N_6_P_2_S_2_ (%): C, 48.42; H, 3.87; N, 8.07; S, 6.16. Found (%): C, 48.62; H, 3.91; N, 7.75; S, 6.55.

## 6. Refinement

Crystal data collection and structure refinement details are summarized in Table 5 ▸. The hydrogen atoms attached to nitro­gen atoms were located from the electron-density map and refined isotropically with distance restraints for complex (**II**). All hydrogen atoms bonded to carbon atoms were placed in calculated idealized positions and refined using a riding model with *U*_iso_(H) = 1.2*U*_eq_(C).

## Supplementary Material

Crystal structure: contains datablock(s) I, II, global. DOI: 10.1107/S2056989026007516/hb8238sup1.cif

Structure factors: contains datablock(s) I. DOI: 10.1107/S2056989026007516/hb8238Isup6.hkl

Structure factors: contains datablock(s) II. DOI: 10.1107/S2056989026007516/hb8238IIsup7.hkl

CCDC references: 2575198, 2575197

Additional supporting information:  crystallographic information; 3D view; checkCIF report

## Figures and Tables

**Table 1:** Selected geometric parameters (Å, °) for (**I**)

Cu1—P1	2.2324 (5)	Cu1—S1	2.3245 (5)
Cu1—Cl1	2.3002 (5)	Cu1—S1^i^	2.5781 (5)
			
P1—Cu1—Cl1	113.502 (19)	Cl1—Cu1—S1^i^	95.874 (17)
P1—Cu1—S1	115.482 (18)	S1—Cu1—S1^i^	109.682 (15)
Cl1—Cu1—S1	112.318 (18)	Cu1—S1—Cu1^i^	70.318 (15)
P1—Cu1—S1^i^	108.064 (18)		

Symmetry code: (i) 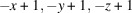.

**Table 2:** Selected geometric parameters (Å, °) for (**II**)

Cu1—P1	2.2376 (6)	Cu1—Br1	2.4284 (4)
Cu1—S1	2.3268 (6)	Cu1—S1^i^	2.5501 (7)
			
P1—Cu1—S1	116.06 (2)	S1—Cu1—S1^i^	109.762 (19)
P1—Cu1—Br1	110.271 (19)	Br1—Cu1—S1^i^	96.366 (17)
S1—Cu1—Br1	113.182 (18)	Cu1—S1—Cu1^i^	70.238 (19)
P1—Cu1—S1^i^	109.40 (2)		

Symmetry code: (i) 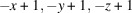.

**Table 3:** Hydrogen-bond geometry (Å, °) for (**I**) *Cg*4 is the centroid of the C10–C15 ring.

*D*—H⋯*A*	*D*—H	H⋯*A*	*D*⋯*A*	*D*—H⋯*A*
N1—H1⋯Cl1	0.83 (2)	2.33 (2)	3.1080 (18)	155.8 (19)
C2—H2⋯Cl1^ii^	0.93	2.79	3.439 (2)	128
C7—H7⋯*Cg*4^iii^	0.93	2.81	3.626 (4)	147

Symmetry codes: (ii) 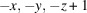; (iii) 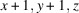.

**Table 4:** Hydrogen-bond geometry (Å, °) for (**II**) *Cg*4 is the centroid of the C10–C15 ring.

*D*—H⋯*A*	*D*—H	H⋯*A*	*D*⋯*A*	*D*—H⋯*A*
N1—H1⋯Br1	0.87 (2)	2.42 (2)	3.235 (2)	156 (2)
C2—H2⋯Br1^ii^	0.93	2.90	3.563 (2)	130
C7—H7⋯*Cg*4^iii^	0.93	2.81	3.638 (4)	149

Symmetry codes: (ii) 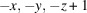; (iii) 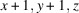.

**Table 5:** Experimental details

	(**I**)	(**II**)
Crystal data		
Chemical formula	[Cu_2_(C_3_H_5_N_3_S)_2_Cl_2_(C_18_H_15_P)_2_]	[Cu_2_(C_3_H_5_N_3_S)_2_Br_2_(C_18_H_15_P)_2_]
*M*_r_	952.84	1041.76
Crystal system, space group	Triclinic, *P* 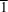	Triclinic, *P* 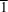
Temperature (K)	296	296
*a*, *b*, *c* (Å)	9.2083 (5), 9.5646 (5), 14.3564 (8)	9.2762 (5), 9.7772 (5), 14.3655 (8)
α, β, γ (°)	103.561 (1), 92.318 (1), 118.208 (1)	103.420 (1), 92.476 (1), 118.188 (1)
*V* (Å^3^)	1066.17 (10)	1099.12 (10)
*Z*	1	1
Radiation type	Mo *K*α	Mo *K*α
μ (mm^−1^)	1.33	2.99
Crystal size (mm)	0.32 × 0.13 × 0.10	0.18 × 0.13 × 0.10

Data collection		
Diffractometer	Bruker APEX CCD area-detector	Bruker APEX CCD area-detector
Absorption correction	Multi-scan (*SADABS*; Krause *et al.*, 2015 ▸)	Multi-scan (*SADABS*; Krause *et al.*, 2015 ▸)
*T*_min_, *T*_max_	0.860, 1.000	0.726, 1.000
No. of measured, independent and observed [*I* > 2σ(*I*)] reflections	15046, 5278, 4507	30282, 5290, 4690
*R*_int_	0.021	0.026
(sin θ/λ)_max_ (Å^−1^)	0.667	0.661

Refinement		
*R*[*F*^2^ > 2σ(*F*^2^)], *wR*(*F*^2^), *S*	0.032, 0.083, 1.03	0.031, 0.076, 1.05
No. of reflections	5278	5290
No. of parameters	258	258
No. of restraints	0	1
H-atom treatment	H atoms treated by a mixture of independent and constrained refinement	H atoms treated by a mixture of independent and constrained refinement
Δρ_max_, Δρ_min_ (e Å^−3^)	0.66, −0.50	0.74, −0.53

Computer programs: *SMART* and *SAINT* (Bruker, 2003 ▸), *SHELXT2018/3* (Sheldrick, 2015*a* ▸), *SHELXL2018/3* (Sheldrick, 2015*b* ▸), *Mercury* (Macrae *et al.*, 2020 ▸), *WinGX* (Farrugia, 2012 ▸) and *publCIF* (Westrip, 2010 ▸).

## supplementary crystallographic information

### Bis(µ-4-methyl-1H-1,2,4-triazole-5-thiolato)bis[chlorido(triphenylphosphane)copper(II)] (I) Crystal data

**Table:**

[Cu_2_(C_3_H_5_N_3_S)_2_Cl_2_(C_18_H_15_P)_2_]	*Z* = 1
*M**_r_* = 952.84	*F*(000) = 488
Triclinic, *P*1	*D*_x_ = 1.484 Mg m^−^^3^
*a* = 9.2083 (5) Å	Mo *K*α radiation, λ = 0.71073 Å
*b* = 9.5646 (5) Å	Cell parameters from 5142 reflections
*c* = 14.3564 (8) Å	θ = 2.5–28.1°
α = 103.561 (1)°	µ = 1.33 mm^−^^1^
β = 92.318 (1)°	*T* = 296 K
γ = 118.208 (1)°	Block, colourless
*V* = 1066.17 (10) Å^3^	0.32 × 0.13 × 0.10 mm

### Bis(µ-4-methyl-1H-1,2,4-triazole-5-thiolato)bis[chlorido(triphenylphosphane)copper(II)] (I) Data collection

**Table:**

Bruker APEX CCD area-detector diffractometer	4507 reflections with *I* > 2σ(*I*)
Radiation source: fine-focus sealed tube	*R*_int_ = 0.021
Frames, each covering 0.3 ° in ω scans	θ_max_ = 28.3°, θ_min_ = 1.5°
Absorption correction: multi-scan (SADABS; Krause *et al.*, 2015)	*h* = −12→12
*T*_min_ = 0.860, *T*_max_ = 1.000	*k* = −12→12
15046 measured reflections	*l* = −19→19
5278 independent reflections	

### Bis(µ-4-methyl-1H-1,2,4-triazole-5-thiolato)bis[chlorido(triphenylphosphane)copper(II)] (I) Refinement

**Table:**

Refinement on *F*^2^	0 restraints
Least-squares matrix: full	Hydrogen site location: mixed
*R*[*F*^2^ > 2σ(*F*^2^)] = 0.032	H atoms treated by a mixture of independent and constrained refinement
*wR*(*F*^2^) = 0.083	*w* = 1/[σ^2^(*F*_o_^2^) + (0.0419*P*)^2^ + 0.3004*P*] where *P* = (*F*_o_^2^ + 2*F*_c_^2^)/3
*S* = 1.03	(Δ/σ)_max_ = 0.001
5278 reflections	Δρ_max_ = 0.66 e Å^−^^3^
258 parameters	Δρ_min_ = −0.50 e Å^−^^3^

### Bis(µ-4-methyl-1H-1,2,4-triazole-5-thiolato)bis[chlorido(triphenylphosphane)copper(II)] (I) Special details

**Table:**

Geometry. All esds (except the esd in the dihedral angle between two l.s. planes) are estimated using the full covariance matrix. The cell esds are taken into account individually in the estimation of esds in distances, angles and torsion angles; correlations between esds in cell parameters are only used when they are defined by crystal symmetry. An approximate (isotropic) treatment of cell esds is used for estimating esds involving l.s. planes.

### Bis(µ-4-methyl-1H-1,2,4-triazole-5-thiolato)bis[chlorido(triphenylphosphane)copper(II)] (I) Fractional atomic coordinates and isotropic or equivalent isotropic displacement parameters (Å^2^)

**Table:**

	*x*	*y*	*z*	*U*_iso_*/*U*_eq_	
Cu1	0.45470 (3)	0.36495 (3)	0.41651 (2)	0.03899 (8)	
Cl1	0.45147 (6)	0.14000 (5)	0.45015 (4)	0.03800 (11)	
S1	0.25331 (5)	0.41894 (6)	0.48179 (3)	0.03421 (11)	
P1	0.46539 (6)	0.36199 (6)	0.26103 (3)	0.03079 (11)	
N1	0.2033 (2)	0.1872 (2)	0.57685 (13)	0.0382 (4)	
N2	0.0905 (2)	0.0875 (2)	0.62506 (13)	0.0457 (4)	
N3	0.00374 (18)	0.23889 (19)	0.57215 (11)	0.0356 (3)	
C1	0.1536 (2)	0.2784 (2)	0.54386 (12)	0.0302 (3)	
C2	−0.0271 (2)	0.1241 (3)	0.62062 (15)	0.0434 (5)	
H2	−0.122763	0.076982	0.647673	0.052*	
C3	−0.1077 (3)	0.2990 (3)	0.54849 (19)	0.0525 (5)	
H3A	−0.116480	0.293619	0.480730	0.079*	
H3B	−0.063635	0.411647	0.587164	0.079*	
H3C	−0.216549	0.231376	0.561966	0.079*	
C4	0.6647 (2)	0.3853 (2)	0.23112 (13)	0.0361 (4)	
C5	0.7146 (3)	0.2824 (3)	0.25912 (16)	0.0495 (5)	
H5	0.645492	0.204952	0.289060	0.059*	
C6	0.8672 (3)	0.2953 (4)	0.24249 (19)	0.0618 (7)	
H6	0.899317	0.225109	0.260256	0.074*	
C7	0.9712 (3)	0.4120 (4)	0.19961 (19)	0.0636 (7)	
H7	1.073705	0.420877	0.188839	0.076*	
C8	0.9241 (3)	0.5143 (4)	0.17303 (19)	0.0639 (7)	
H8	0.994978	0.592659	0.144147	0.077*	
C9	0.7712 (3)	0.5029 (3)	0.18860 (16)	0.0480 (5)	
H9	0.740429	0.573759	0.170576	0.058*	
C10	0.4354 (2)	0.5133 (2)	0.21824 (13)	0.0323 (4)	
C11	0.4370 (2)	0.6428 (2)	0.28772 (14)	0.0384 (4)	
H11	0.449372	0.647418	0.353168	0.046*	
C12	0.4203 (3)	0.7650 (3)	0.26076 (18)	0.0529 (5)	
H12	0.425226	0.853073	0.308020	0.063*	
C13	0.3965 (3)	0.7558 (3)	0.1645 (2)	0.0617 (6)	
H13	0.385835	0.838188	0.146377	0.074*	
C14	0.3881 (3)	0.6251 (3)	0.09386 (18)	0.0602 (6)	
H14	0.368879	0.618120	0.028394	0.072*	
C15	0.4082 (3)	0.5046 (3)	0.12038 (15)	0.0452 (5)	
H15	0.403477	0.417266	0.072648	0.054*	
C16	0.3106 (2)	0.1657 (2)	0.17376 (14)	0.0365 (4)	
C17	0.3434 (3)	0.0950 (3)	0.08679 (16)	0.0493 (5)	
H17	0.450010	0.145782	0.071450	0.059*	
C18	0.2170 (4)	−0.0512 (3)	0.02293 (19)	0.0654 (7)	
H18	0.238580	−0.097701	−0.035577	0.078*	
C19	0.0606 (4)	−0.1272 (3)	0.0458 (2)	0.0728 (8)	
H19	−0.023333	−0.225805	0.003076	0.087*	
C20	0.0267 (3)	−0.0590 (3)	0.1313 (2)	0.0700 (8)	
H20	−0.080403	−0.110475	0.145972	0.084*	
C21	0.1520 (3)	0.0867 (3)	0.19603 (17)	0.0498 (5)	
H21	0.129383	0.131575	0.254638	0.060*	
H1	0.281 (3)	0.173 (3)	0.5592 (15)	0.036 (5)*	

### Bis(µ-4-methyl-1H-1,2,4-triazole-5-thiolato)bis[chlorido(triphenylphosphane)copper(II)] (I) Atomic displacement parameters (Å^2^)

**Table:**

	*U*^11^	*U*^22^	*U*^33^	*U*^12^	*U*^13^	*U*^23^
Cu1	0.05047 (15)	0.04642 (15)	0.03399 (13)	0.03140 (12)	0.01482 (10)	0.01793 (10)
Cl1	0.0379 (2)	0.0318 (2)	0.0493 (3)	0.02040 (18)	0.00945 (19)	0.01311 (19)
S1	0.0319 (2)	0.0366 (2)	0.0415 (2)	0.01964 (19)	0.01161 (18)	0.01718 (19)
P1	0.0334 (2)	0.0362 (2)	0.0282 (2)	0.02067 (19)	0.00782 (17)	0.01070 (18)
N1	0.0323 (8)	0.0397 (9)	0.0464 (9)	0.0176 (7)	0.0107 (7)	0.0190 (7)
N2	0.0398 (9)	0.0447 (9)	0.0520 (10)	0.0156 (8)	0.0108 (7)	0.0245 (8)
N3	0.0272 (7)	0.0374 (8)	0.0379 (8)	0.0128 (6)	0.0075 (6)	0.0102 (7)
C1	0.0260 (8)	0.0296 (8)	0.0308 (8)	0.0121 (7)	0.0041 (6)	0.0054 (7)
C2	0.0335 (9)	0.0443 (11)	0.0455 (11)	0.0117 (8)	0.0102 (8)	0.0173 (9)
C3	0.0368 (10)	0.0645 (14)	0.0697 (15)	0.0315 (10)	0.0179 (10)	0.0270 (12)
C4	0.0348 (9)	0.0443 (10)	0.0301 (9)	0.0224 (8)	0.0064 (7)	0.0060 (8)
C5	0.0496 (12)	0.0617 (13)	0.0524 (13)	0.0370 (11)	0.0152 (10)	0.0205 (11)
C6	0.0568 (14)	0.0818 (18)	0.0621 (15)	0.0508 (14)	0.0072 (12)	0.0111 (13)
C7	0.0372 (11)	0.0853 (19)	0.0593 (15)	0.0328 (12)	0.0087 (10)	−0.0001 (13)
C8	0.0414 (12)	0.0753 (17)	0.0663 (16)	0.0216 (12)	0.0220 (11)	0.0197 (13)
C9	0.0395 (10)	0.0522 (12)	0.0508 (12)	0.0212 (9)	0.0112 (9)	0.0152 (10)
C10	0.0310 (8)	0.0353 (9)	0.0325 (9)	0.0170 (7)	0.0063 (7)	0.0115 (7)
C11	0.0382 (9)	0.0381 (10)	0.0373 (10)	0.0195 (8)	0.0051 (8)	0.0071 (8)
C12	0.0592 (13)	0.0385 (11)	0.0649 (15)	0.0282 (10)	0.0130 (11)	0.0126 (10)
C13	0.0758 (16)	0.0567 (14)	0.0763 (17)	0.0430 (13)	0.0196 (13)	0.0364 (13)
C14	0.0803 (17)	0.0720 (16)	0.0493 (13)	0.0468 (14)	0.0155 (12)	0.0331 (12)
C15	0.0609 (13)	0.0520 (12)	0.0347 (10)	0.0355 (11)	0.0128 (9)	0.0156 (9)
C16	0.0417 (10)	0.0325 (9)	0.0378 (10)	0.0198 (8)	0.0023 (8)	0.0120 (8)
C17	0.0579 (13)	0.0468 (12)	0.0435 (11)	0.0282 (10)	0.0053 (10)	0.0090 (9)
C18	0.0844 (19)	0.0522 (14)	0.0479 (13)	0.0341 (14)	−0.0100 (13)	−0.0024 (11)
C19	0.0754 (18)	0.0426 (13)	0.0695 (18)	0.0128 (13)	−0.0243 (15)	0.0055 (12)
C20	0.0493 (14)	0.0553 (15)	0.085 (2)	0.0073 (12)	−0.0041 (13)	0.0297 (15)
C21	0.0443 (11)	0.0475 (12)	0.0548 (13)	0.0187 (10)	0.0059 (9)	0.0194 (10)

### Bis(µ-4-methyl-1H-1,2,4-triazole-5-thiolato)bis[chlorido(triphenylphosphane)copper(II)] (I) Geometric parameters (Å, º)

**Table:**

Cu1—P1	2.2324 (5)	C7—H7	0.9300
Cu1—Cl1	2.3002 (5)	C8—C9	1.392 (3)
Cu1—S1	2.3245 (5)	C8—H8	0.9300
Cu1—S1^i^	2.5781 (5)	C9—H9	0.9300
Cu1—Cu1^i^	2.8308 (5)	C10—C11	1.390 (3)
S1—C1	1.7042 (18)	C10—C15	1.393 (3)
P1—C4	1.8236 (18)	C11—C12	1.385 (3)
P1—C10	1.8249 (18)	C11—H11	0.9300
P1—C16	1.8299 (19)	C12—C13	1.366 (4)
N1—C1	1.323 (2)	C12—H12	0.9300
N1—N2	1.378 (2)	C13—C14	1.380 (4)
N1—H1	0.83 (2)	C13—H13	0.9300
N2—C2	1.289 (3)	C14—C15	1.381 (3)
N3—C1	1.355 (2)	C14—H14	0.9300
N3—C2	1.360 (3)	C15—H15	0.9300
N3—C3	1.456 (3)	C16—C21	1.383 (3)
C2—H2	0.9300	C16—C17	1.391 (3)
C3—H3A	0.9600	C17—C18	1.388 (3)
C3—H3B	0.9600	C17—H17	0.9300
C3—H3C	0.9600	C18—C19	1.368 (4)
C4—C9	1.388 (3)	C18—H18	0.9300
C4—C5	1.392 (3)	C19—C20	1.370 (4)
C5—C6	1.387 (3)	C19—H19	0.9300
C5—H5	0.9300	C20—C21	1.387 (3)
C6—C7	1.379 (4)	C20—H20	0.9300
C6—H6	0.9300	C21—H21	0.9300
C7—C8	1.361 (4)		
			
P1—Cu1—Cl1	113.502 (19)	C5—C6—H6	120.0
P1—Cu1—S1	115.482 (18)	C8—C7—C6	120.2 (2)
Cl1—Cu1—S1	112.318 (18)	C8—C7—H7	119.9
P1—Cu1—S1^i^	108.064 (18)	C6—C7—H7	119.9
Cl1—Cu1—S1^i^	95.874 (17)	C7—C8—C9	120.7 (2)
S1—Cu1—S1^i^	109.682 (15)	C7—C8—H8	119.6
P1—Cu1—Cu1^i^	129.471 (18)	C9—C8—H8	119.6
Cl1—Cu1—Cu1^i^	113.892 (17)	C4—C9—C8	119.8 (2)
S1—Cu1—Cu1^i^	59.042 (14)	C4—C9—H9	120.1
S1^i^—Cu1—Cu1^i^	50.639 (13)	C8—C9—H9	120.1
C1—S1—Cu1	107.31 (6)	C11—C10—C15	118.44 (17)
C1—S1—Cu1^i^	107.96 (6)	C11—C10—P1	117.70 (14)
Cu1—S1—Cu1^i^	70.318 (15)	C15—C10—P1	123.84 (15)
C4—P1—C10	106.20 (8)	C12—C11—C10	120.85 (19)
C4—P1—C16	103.39 (9)	C12—C11—H11	119.6
C10—P1—C16	102.43 (8)	C10—C11—H11	119.6
C4—P1—Cu1	110.54 (6)	C13—C12—C11	119.8 (2)
C10—P1—Cu1	118.81 (6)	C13—C12—H12	120.1
C16—P1—Cu1	114.02 (6)	C11—C12—H12	120.1
C1—N1—N2	113.14 (16)	C12—C13—C14	120.5 (2)
C1—N1—H1	123.6 (15)	C12—C13—H13	119.8
N2—N1—H1	121.7 (15)	C14—C13—H13	119.8
C2—N2—N1	102.62 (16)	C13—C14—C15	120.0 (2)
C1—N3—C2	106.78 (16)	C13—C14—H14	120.0
C1—N3—C3	126.20 (17)	C15—C14—H14	120.0
C2—N3—C3	126.87 (17)	C14—C15—C10	120.4 (2)
N1—C1—N3	104.63 (15)	C14—C15—H15	119.8
N1—C1—S1	129.03 (13)	C10—C15—H15	119.8
N3—C1—S1	126.33 (14)	C21—C16—C17	119.12 (19)
N2—C2—N3	112.83 (17)	C21—C16—P1	117.20 (16)
N2—C2—H2	123.6	C17—C16—P1	123.66 (16)
N3—C2—H2	123.6	C18—C17—C16	120.0 (2)
N3—C3—H3A	109.5	C18—C17—H17	120.0
N3—C3—H3B	109.5	C16—C17—H17	120.0
H3A—C3—H3B	109.5	C19—C18—C17	120.1 (3)
N3—C3—H3C	109.5	C19—C18—H18	119.9
H3A—C3—H3C	109.5	C17—C18—H18	119.9
H3B—C3—H3C	109.5	C18—C19—C20	120.5 (2)
C9—C4—C5	119.14 (18)	C18—C19—H19	119.8
C9—C4—P1	124.71 (16)	C20—C19—H19	119.8
C5—C4—P1	116.03 (15)	C19—C20—C21	120.0 (3)
C6—C5—C4	120.2 (2)	C19—C20—H20	120.0
C6—C5—H5	119.9	C21—C20—H20	120.0
C4—C5—H5	119.9	C16—C21—C20	120.3 (2)
C7—C6—C5	120.0 (2)	C16—C21—H21	119.9
C7—C6—H6	120.0	C20—C21—H21	119.9
			
C1—N1—N2—C2	−0.7 (2)	C4—P1—C10—C11	−113.94 (15)
N2—N1—C1—N3	0.6 (2)	C16—P1—C10—C11	137.94 (15)
N2—N1—C1—S1	179.34 (14)	Cu1—P1—C10—C11	11.29 (16)
C2—N3—C1—N1	−0.3 (2)	C4—P1—C10—C15	67.28 (18)
C3—N3—C1—N1	−176.07 (18)	C16—P1—C10—C15	−40.84 (18)
C2—N3—C1—S1	−179.03 (14)	Cu1—P1—C10—C15	−167.50 (14)
C3—N3—C1—S1	5.2 (3)	C15—C10—C11—C12	−3.2 (3)
Cu1—S1—C1—N1	18.99 (18)	P1—C10—C11—C12	177.95 (16)
Cu1^i^—S1—C1—N1	−55.36 (18)	C10—C11—C12—C13	2.2 (3)
Cu1—S1—C1—N3	−162.55 (14)	C11—C12—C13—C14	0.3 (4)
Cu1^i^—S1—C1—N3	123.10 (15)	C12—C13—C14—C15	−1.7 (4)
N1—N2—C2—N3	0.5 (2)	C13—C14—C15—C10	0.6 (4)
C1—N3—C2—N2	−0.2 (2)	C11—C10—C15—C14	1.8 (3)
C3—N3—C2—N2	175.6 (2)	P1—C10—C15—C14	−179.41 (18)
C10—P1—C4—C9	2.9 (2)	C4—P1—C16—C21	160.08 (15)
C16—P1—C4—C9	110.31 (18)	C10—P1—C16—C21	−89.67 (16)
Cu1—P1—C4—C9	−127.27 (16)	Cu1—P1—C16—C21	40.01 (17)
C10—P1—C4—C5	178.92 (15)	C4—P1—C16—C17	−21.18 (19)
C16—P1—C4—C5	−73.65 (16)	C10—P1—C16—C17	89.07 (18)
Cu1—P1—C4—C5	48.78 (17)	Cu1—P1—C16—C17	−141.25 (15)
C9—C4—C5—C6	−1.5 (3)	C21—C16—C17—C18	1.1 (3)
P1—C4—C5—C6	−177.77 (18)	P1—C16—C17—C18	−177.57 (17)
C4—C5—C6—C7	1.1 (4)	C16—C17—C18—C19	−0.8 (4)
C5—C6—C7—C8	−0.4 (4)	C17—C18—C19—C20	0.7 (4)
C6—C7—C8—C9	0.0 (4)	C18—C19—C20—C21	−1.0 (4)
C5—C4—C9—C8	1.1 (3)	C17—C16—C21—C20	−1.4 (3)
P1—C4—C9—C8	177.08 (18)	P1—C16—C21—C20	177.38 (18)
C7—C8—C9—C4	−0.4 (4)	C19—C20—C21—C16	1.3 (4)

Symmetry code: (i) −*x*+1, −*y*+1, −*z*+1.

### Bis(µ-4-methyl-1H-1,2,4-triazole-5-thiolato)bis[chlorido(triphenylphosphane)copper(II)] (I) Hydrogen-bond geometry (Å, º)

Cg4 is the centroid of the C10–C15 ring.

**Table:**

*D*—H···*A*	*D*—H	H···*A*	*D*···*A*	*D*—H···*A*
N1—H1···Cl1	0.83 (2)	2.33 (2)	3.1080 (18)	155.8 (19)
C2—H2···Cl1^ii^	0.93	2.79	3.439 (2)	128
C7—H7···*Cg*4^iii^	0.93	2.81	3.626 (4)	147

Symmetry codes: (ii) −*x*, −*y*, −*z*+1; (iii) *x*+1, *y*+1, *z*.

### Bis(µ-4-methyl-1H-1,2,4-triazole-5-thiolato)bis[bromido(triphenylphosphane)copper(II)] (II) Crystal data

**Table:**

[Cu_2_(C_3_H_5_N_3_S)_2_Br_2_(C_18_H_15_P)_2_]	*Z* = 1
*M**_r_* = 1041.76	*F*(000) = 524
Triclinic, *P*1	*D*_x_ = 1.574 Mg m^−^^3^
*a* = 9.2762 (5) Å	Mo *K*α radiation, λ = 0.71073 Å
*b* = 9.7772 (5) Å	Cell parameters from 4760 reflections
*c* = 14.3655 (8) Å	θ = 2.5–27.0°
α = 103.420 (1)°	µ = 2.99 mm^−^^1^
β = 92.476 (1)°	*T* = 296 K
γ = 118.188 (1)°	Block, colourless
*V* = 1099.12 (10) Å^3^	0.18 × 0.13 × 0.10 mm

### Bis(µ-4-methyl-1H-1,2,4-triazole-5-thiolato)bis[bromido(triphenylphosphane)copper(II)] (II) Data collection

**Table:**

Bruker APEX CCD area-detector diffractometer	4690 reflections with *I* > 2σ(*I*)
Radiation source: fine-focus sealed tube	*R*_int_ = 0.026
Frames, each covering 0.3 ° in ω scans	θ_max_ = 28.0°, θ_min_ = 1.5°
Absorption correction: multi-scan (SADABS; Krause *et al.*, 2015)	*h* = −12→12
*T*_min_ = 0.726, *T*_max_ = 1.000	*k* = −12→12
30282 measured reflections	*l* = −18→18
5290 independent reflections	

### Bis(µ-4-methyl-1H-1,2,4-triazole-5-thiolato)bis[bromido(triphenylphosphane)copper(II)] (II) Refinement

**Table:**

Refinement on *F*^2^	1 restraint
Least-squares matrix: full	Hydrogen site location: mixed
*R*[*F*^2^ > 2σ(*F*^2^)] = 0.031	H atoms treated by a mixture of independent and constrained refinement
*wR*(*F*^2^) = 0.076	*w* = 1/[σ^2^(*F*_o_^2^) + (0.0332*P*)^2^ + 0.7926*P*] where *P* = (*F*_o_^2^ + 2*F*_c_^2^)/3
*S* = 1.05	(Δ/σ)_max_ = 0.001
5290 reflections	Δρ_max_ = 0.74 e Å^−^^3^
258 parameters	Δρ_min_ = −0.53 e Å^−^^3^

### Bis(µ-4-methyl-1H-1,2,4-triazole-5-thiolato)bis[bromido(triphenylphosphane)copper(II)] (II) Special details

**Table:**

Geometry. All esds (except the esd in the dihedral angle between two l.s. planes) are estimated using the full covariance matrix. The cell esds are taken into account individually in the estimation of esds in distances, angles and torsion angles; correlations between esds in cell parameters are only used when they are defined by crystal symmetry. An approximate (isotropic) treatment of cell esds is used for estimating esds involving l.s. planes.

### Bis(µ-4-methyl-1H-1,2,4-triazole-5-thiolato)bis[bromido(triphenylphosphane)copper(II)] (II) Fractional atomic coordinates and isotropic or equivalent isotropic displacement parameters (Å^2^)

**Table:**

	*x*	*y*	*z*	*U*_iso_*/*U*_eq_	
Cu1	0.45873 (4)	0.37211 (4)	0.41591 (2)	0.04068 (8)	
Br1	0.45899 (3)	0.13662 (3)	0.44388 (2)	0.04157 (7)	
S1	0.25583 (7)	0.41875 (7)	0.48190 (4)	0.03694 (12)	
P1	0.46591 (7)	0.36575 (7)	0.25953 (4)	0.03364 (12)	
N1	0.2049 (3)	0.1890 (3)	0.57543 (16)	0.0434 (4)	
N2	0.0933 (3)	0.0923 (3)	0.62374 (17)	0.0522 (5)	
N3	0.0100 (2)	0.2438 (2)	0.57337 (14)	0.0402 (4)	
C1	0.1576 (3)	0.2809 (3)	0.54395 (15)	0.0341 (4)	
C2	−0.0215 (3)	0.1307 (3)	0.62101 (19)	0.0493 (6)	
H2	−0.115806	0.085686	0.648695	0.059*	
C3	−0.0971 (3)	0.3089 (4)	0.5529 (3)	0.0620 (8)	
H3A	−0.121376	0.290030	0.484005	0.093*	
H3B	−0.041336	0.423162	0.584681	0.093*	
H3C	−0.198766	0.256126	0.576615	0.093*	
C4	0.6634 (3)	0.3905 (3)	0.22766 (16)	0.0400 (5)	
C5	0.7133 (4)	0.2863 (4)	0.2506 (2)	0.0561 (7)	
H5	0.644763	0.206714	0.278193	0.067*	
C6	0.8648 (4)	0.3010 (5)	0.2323 (2)	0.0672 (8)	
H6	0.896827	0.230381	0.246886	0.081*	
C7	0.9675 (4)	0.4193 (4)	0.1929 (2)	0.0677 (9)	
H7	1.068841	0.428532	0.180602	0.081*	
C8	0.9216 (4)	0.5236 (4)	0.1716 (2)	0.0673 (8)	
H8	0.992237	0.604263	0.145300	0.081*	
C9	0.7694 (3)	0.5098 (4)	0.1890 (2)	0.0521 (6)	
H9	0.739135	0.581630	0.174412	0.063*	
C10	0.4330 (3)	0.5114 (3)	0.21502 (16)	0.0354 (4)	
C11	0.4350 (3)	0.6386 (3)	0.28340 (18)	0.0433 (5)	
H11	0.449233	0.644998	0.349061	0.052*	
C12	0.4162 (4)	0.7560 (3)	0.2553 (2)	0.0582 (7)	
H12	0.420455	0.842247	0.301857	0.070*	
C13	0.3910 (4)	0.7446 (4)	0.1582 (3)	0.0671 (8)	
H13	0.380165	0.824414	0.139116	0.081*	
C14	0.3819 (4)	0.6159 (4)	0.0892 (2)	0.0627 (8)	
H14	0.361021	0.607044	0.023583	0.075*	
C15	0.4034 (3)	0.4995 (3)	0.11675 (18)	0.0483 (6)	
H15	0.398143	0.413249	0.069674	0.058*	
C16	0.3119 (3)	0.1715 (3)	0.17498 (17)	0.0413 (5)	
C17	0.3427 (4)	0.1003 (3)	0.0884 (2)	0.0558 (7)	
H17	0.447284	0.150032	0.071603	0.067*	
C18	0.2163 (5)	−0.0456 (4)	0.0269 (2)	0.0741 (10)	
H18	0.236950	−0.093564	−0.031009	0.089*	
C19	0.0623 (5)	−0.1189 (4)	0.0509 (3)	0.0820 (12)	
H19	−0.021660	−0.216111	0.009202	0.098*	
C20	0.0310 (4)	−0.0497 (4)	0.1361 (3)	0.0764 (10)	
H20	−0.074323	−0.099934	0.151897	0.092*	
C21	0.1551 (3)	0.0944 (3)	0.1989 (2)	0.0554 (7)	
H21	0.133495	0.139842	0.257166	0.066*	
H1	0.286 (3)	0.175 (3)	0.5569 (18)	0.037 (6)*	

### Bis(µ-4-methyl-1H-1,2,4-triazole-5-thiolato)bis[bromido(triphenylphosphane)copper(II)] (II) Atomic displacement parameters (Å^2^)

**Table:**

	*U*^11^	*U*^22^	*U*^33^	*U*^12^	*U*^13^	*U*^23^
Cu1	0.05287 (17)	0.04652 (17)	0.03529 (15)	0.03174 (14)	0.01301 (12)	0.01691 (12)
Br1	0.04191 (13)	0.03418 (12)	0.05408 (15)	0.02244 (10)	0.00859 (10)	0.01417 (10)
S1	0.0346 (3)	0.0394 (3)	0.0437 (3)	0.0212 (2)	0.0104 (2)	0.0169 (2)
P1	0.0375 (3)	0.0385 (3)	0.0303 (3)	0.0227 (2)	0.0072 (2)	0.0104 (2)
N1	0.0371 (10)	0.0468 (11)	0.0527 (12)	0.0216 (9)	0.0123 (9)	0.0227 (9)
N2	0.0451 (12)	0.0494 (12)	0.0591 (14)	0.0152 (10)	0.0111 (10)	0.0286 (11)
N3	0.0293 (9)	0.0435 (11)	0.0423 (11)	0.0145 (8)	0.0060 (8)	0.0111 (9)
C1	0.0285 (10)	0.0330 (10)	0.0334 (10)	0.0122 (8)	0.0018 (8)	0.0044 (8)
C2	0.0385 (12)	0.0504 (14)	0.0489 (14)	0.0121 (11)	0.0105 (10)	0.0189 (11)
C3	0.0409 (14)	0.079 (2)	0.080 (2)	0.0362 (14)	0.0187 (13)	0.0302 (17)
C4	0.0390 (11)	0.0494 (13)	0.0331 (11)	0.0253 (10)	0.0056 (9)	0.0071 (9)
C5	0.0578 (16)	0.0678 (18)	0.0611 (17)	0.0426 (15)	0.0184 (13)	0.0233 (14)
C6	0.0623 (18)	0.087 (2)	0.0688 (19)	0.0545 (18)	0.0097 (15)	0.0131 (17)
C7	0.0437 (15)	0.090 (2)	0.0646 (19)	0.0369 (16)	0.0111 (13)	0.0050 (17)
C8	0.0423 (15)	0.078 (2)	0.073 (2)	0.0240 (15)	0.0171 (14)	0.0202 (17)
C9	0.0436 (13)	0.0591 (16)	0.0534 (15)	0.0249 (12)	0.0120 (11)	0.0163 (13)
C10	0.0365 (11)	0.0407 (11)	0.0340 (11)	0.0222 (9)	0.0074 (8)	0.0122 (9)
C11	0.0459 (13)	0.0401 (12)	0.0417 (12)	0.0220 (11)	0.0035 (10)	0.0072 (10)
C12	0.0668 (18)	0.0435 (14)	0.0700 (19)	0.0337 (14)	0.0132 (14)	0.0118 (13)
C13	0.081 (2)	0.0636 (18)	0.082 (2)	0.0475 (17)	0.0206 (17)	0.0384 (17)
C14	0.084 (2)	0.080 (2)	0.0483 (15)	0.0519 (18)	0.0178 (14)	0.0357 (15)
C15	0.0628 (16)	0.0581 (15)	0.0363 (12)	0.0380 (13)	0.0126 (11)	0.0161 (11)
C16	0.0472 (13)	0.0385 (12)	0.0398 (12)	0.0226 (10)	0.0000 (10)	0.0124 (9)
C17	0.0675 (18)	0.0514 (15)	0.0472 (14)	0.0327 (14)	0.0029 (13)	0.0057 (12)
C18	0.098 (3)	0.0552 (18)	0.0558 (18)	0.0387 (19)	−0.0143 (17)	−0.0039 (14)
C19	0.088 (3)	0.0470 (17)	0.080 (2)	0.0178 (18)	−0.031 (2)	0.0075 (17)
C20	0.0567 (18)	0.0612 (19)	0.088 (3)	0.0086 (15)	−0.0081 (17)	0.0306 (19)
C21	0.0486 (14)	0.0540 (16)	0.0600 (17)	0.0205 (13)	0.0048 (12)	0.0223 (13)

### Bis(µ-4-methyl-1H-1,2,4-triazole-5-thiolato)bis[bromido(triphenylphosphane)copper(II)] (II) Geometric parameters (Å, º)

**Table:**

Cu1—P1	2.2376 (6)	C7—H7	0.9300
Cu1—S1	2.3268 (6)	C8—C9	1.393 (4)
Cu1—Br1	2.4284 (4)	C8—H8	0.9300
Cu1—S1^i^	2.5501 (7)	C9—H9	0.9300
Cu1—Cu1^i^	2.8115 (6)	C10—C11	1.386 (3)
S1—C1	1.706 (2)	C10—C15	1.394 (3)
P1—C4	1.824 (2)	C11—C12	1.382 (4)
P1—C16	1.827 (2)	C11—H11	0.9300
P1—C10	1.829 (2)	C12—C13	1.374 (5)
N1—C1	1.323 (3)	C12—H12	0.9300
N1—N2	1.374 (3)	C13—C14	1.373 (5)
N1—H1	0.873 (16)	C13—H13	0.9300
N2—C2	1.287 (4)	C14—C15	1.381 (4)
N3—C1	1.355 (3)	C14—H14	0.9300
N3—C2	1.356 (3)	C15—H15	0.9300
N3—C3	1.461 (3)	C16—C21	1.389 (4)
C2—H2	0.9300	C16—C17	1.389 (4)
C3—H3A	0.9600	C17—C18	1.391 (4)
C3—H3B	0.9600	C17—H17	0.9300
C3—H3C	0.9600	C18—C19	1.366 (6)
C4—C9	1.379 (4)	C18—H18	0.9300
C4—C5	1.396 (4)	C19—C20	1.368 (6)
C5—C6	1.386 (4)	C19—H19	0.9300
C5—H5	0.9300	C20—C21	1.383 (4)
C6—C7	1.369 (5)	C20—H20	0.9300
C6—H6	0.9300	C21—H21	0.9300
C7—C8	1.363 (5)		
			
P1—Cu1—S1	116.06 (2)	C5—C6—H6	119.9
P1—Cu1—Br1	110.271 (19)	C8—C7—C6	120.2 (3)
S1—Cu1—Br1	113.182 (18)	C8—C7—H7	119.9
P1—Cu1—S1^i^	109.40 (2)	C6—C7—H7	119.9
S1—Cu1—S1^i^	109.762 (19)	C7—C8—C9	120.3 (3)
Br1—Cu1—S1^i^	96.366 (17)	C7—C8—H8	119.9
P1—Cu1—Cu1^i^	131.67 (2)	C9—C8—H8	119.9
S1—Cu1—Cu1^i^	58.606 (18)	C4—C9—C8	120.5 (3)
Br1—Cu1—Cu1^i^	115.238 (16)	C4—C9—H9	119.8
S1^i^—Cu1—Cu1^i^	51.156 (16)	C8—C9—H9	119.8
C1—S1—Cu1	108.45 (8)	C11—C10—C15	118.7 (2)
C1—S1—Cu1^i^	107.43 (7)	C11—C10—P1	117.67 (17)
Cu1—S1—Cu1^i^	70.238 (19)	C15—C10—P1	123.67 (18)
C4—P1—C16	103.53 (11)	C12—C11—C10	120.9 (2)
C4—P1—C10	105.42 (11)	C12—C11—H11	119.6
C16—P1—C10	102.79 (10)	C10—C11—H11	119.6
C4—P1—Cu1	111.26 (8)	C13—C12—C11	119.7 (3)
C16—P1—Cu1	113.44 (8)	C13—C12—H12	120.1
C10—P1—Cu1	118.92 (7)	C11—C12—H12	120.1
C1—N1—N2	113.2 (2)	C14—C13—C12	120.2 (3)
C1—N1—H1	123.6 (17)	C14—C13—H13	119.9
N2—N1—H1	121.9 (17)	C12—C13—H13	119.9
C2—N2—N1	102.7 (2)	C13—C14—C15	120.4 (3)
C1—N3—C2	107.0 (2)	C13—C14—H14	119.8
C1—N3—C3	125.8 (2)	C15—C14—H14	119.8
C2—N3—C3	127.1 (2)	C14—C15—C10	120.0 (2)
N1—C1—N3	104.35 (19)	C14—C15—H15	120.0
N1—C1—S1	129.51 (17)	C10—C15—H15	120.0
N3—C1—S1	126.13 (17)	C21—C16—C17	119.1 (3)
N2—C2—N3	112.7 (2)	C21—C16—P1	117.3 (2)
N2—C2—H2	123.6	C17—C16—P1	123.6 (2)
N3—C2—H2	123.6	C16—C17—C18	119.7 (3)
N3—C3—H3A	109.5	C16—C17—H17	120.1
N3—C3—H3B	109.5	C18—C17—H17	120.1
H3A—C3—H3B	109.5	C19—C18—C17	120.4 (3)
N3—C3—H3C	109.5	C19—C18—H18	119.8
H3A—C3—H3C	109.5	C17—C18—H18	119.8
H3B—C3—H3C	109.5	C18—C19—C20	120.2 (3)
C9—C4—C5	118.6 (2)	C18—C19—H19	119.9
C9—C4—P1	124.8 (2)	C20—C19—H19	119.9
C5—C4—P1	116.5 (2)	C19—C20—C21	120.4 (3)
C6—C5—C4	120.2 (3)	C19—C20—H20	119.8
C6—C5—H5	119.9	C21—C20—H20	119.8
C4—C5—H5	119.9	C20—C21—C16	120.1 (3)
C7—C6—C5	120.2 (3)	C20—C21—H21	119.9
C7—C6—H6	119.9	C16—C21—H21	119.9
			
C1—N1—N2—C2	−0.6 (3)	C4—P1—C10—C11	−114.09 (19)
N2—N1—C1—N3	0.4 (3)	C16—P1—C10—C11	137.76 (19)
N2—N1—C1—S1	179.53 (18)	Cu1—P1—C10—C11	11.5 (2)
C2—N3—C1—N1	0.0 (2)	C4—P1—C10—C15	67.1 (2)
C3—N3—C1—N1	−177.4 (2)	C16—P1—C10—C15	−41.1 (2)
C2—N3—C1—S1	−179.18 (18)	Cu1—P1—C10—C15	−167.33 (18)
C3—N3—C1—S1	3.5 (3)	C15—C10—C11—C12	−3.1 (4)
Cu1—S1—C1—N1	17.2 (2)	P1—C10—C11—C12	178.0 (2)
Cu1^i^—S1—C1—N1	−57.3 (2)	C10—C11—C12—C13	1.6 (4)
Cu1—S1—C1—N3	−163.92 (17)	C11—C12—C13—C14	1.1 (5)
Cu1^i^—S1—C1—N3	121.68 (18)	C12—C13—C14—C15	−2.2 (5)
N1—N2—C2—N3	0.6 (3)	C13—C14—C15—C10	0.6 (5)
C1—N3—C2—N2	−0.4 (3)	C11—C10—C15—C14	2.0 (4)
C3—N3—C2—N2	176.9 (3)	P1—C10—C15—C14	−179.2 (2)
C16—P1—C4—C9	114.6 (2)	C4—P1—C16—C21	160.9 (2)
C10—P1—C4—C9	7.0 (2)	C10—P1—C16—C21	−89.5 (2)
Cu1—P1—C4—C9	−123.2 (2)	Cu1—P1—C16—C21	40.2 (2)
C16—P1—C4—C5	−69.7 (2)	C4—P1—C16—C17	−20.6 (2)
C10—P1—C4—C5	−177.3 (2)	C10—P1—C16—C17	89.0 (2)
Cu1—P1—C4—C5	52.5 (2)	Cu1—P1—C16—C17	−141.3 (2)
C9—C4—C5—C6	−1.6 (4)	C21—C16—C17—C18	0.5 (4)
P1—C4—C5—C6	−177.5 (2)	P1—C16—C17—C18	−178.0 (2)
C4—C5—C6—C7	0.9 (5)	C16—C17—C18—C19	0.2 (5)
C5—C6—C7—C8	0.2 (5)	C17—C18—C19—C20	−0.4 (5)
C6—C7—C8—C9	−0.5 (5)	C18—C19—C20—C21	−0.3 (5)
C5—C4—C9—C8	1.2 (4)	C19—C20—C21—C16	1.1 (5)
P1—C4—C9—C8	176.8 (2)	C17—C16—C21—C20	−1.2 (4)
C7—C8—C9—C4	−0.2 (5)	P1—C16—C21—C20	177.5 (2)

Symmetry code: (i) −*x*+1, −*y*+1, −*z*+1.

### Bis(µ-4-methyl-1H-1,2,4-triazole-5-thiolato)bis[bromido(triphenylphosphane)copper(II)] (II) Hydrogen-bond geometry (Å, º)

Cg4 is the centroid of the C10–C15 ring.

**Table:**

*D*—H···*A*	*D*—H	H···*A*	*D*···*A*	*D*—H···*A*
N1—H1···Br1	0.87 (2)	2.42 (2)	3.235 (2)	156 (2)
C2—H2···Br1^ii^	0.93	2.90	3.563 (2)	130
C7—H7···*Cg*4^iii^	0.93	2.81	3.638 (4)	149

Symmetry codes: (ii) −*x*, −*y*, −*z*+1; (iii) *x*+1, *y*+1, *z*.
